# Evaluation of Certain Pharmaceutical Quality Attributes of Lisinopril Split Tablets

**DOI:** 10.3390/scipharm84040646

**Published:** 2016-10-11

**Authors:** Khairi M. S. Fahelelbom, Moawia M. M. Al-Tabakha, Nermin A. M. Eissa, Jeevani Javadi

**Affiliations:** Pharmaceutical Sciences Unit, College of Pharmacy, Al Ain University of Science and Technology, P.O. Box 64141 Al Ain, UAE; nermin.abdelwahab@aau.ac.ae (N.A.M.E.); Jeevani.Javadi@aau.ac.ae (J.J.); Department of Pharmaceutics, College of Pharmacy and Health Sciences, Ajman University of Sciences and Technology, P.O. Box 346 Ajman, UAE; M.Altabakha@Ajman.ac.ae

**Keywords:** tablet splitting, lisinopril, weight variation, disintegration, dissolution spectrophotometric analysis

## Abstract

Tablet splitting is an accepted practice for the administration of drugs for a variety of reasons, including dose adjustment, ease of swallowing and cost savings. The purpose of this study was to evaluate the physical properties of lisinopril tablets as a result of splitting the tablets either by hand or with a splitting device. The impact of the splitting technique of lisinopril (Zestril^®^ tablets, 20 mg) on certain physical parameters such as weight variation, friability, disintegration, dissolution and drug content were studied. Splitting the tablets either by hand or with a splitter resulted in a minute but statistically significant average weight loss of <0.25% of the tablet to the surrounding environment. The variability in the weight of the hand-split tablet halves was more pronounced (37 out of 40 tablet halves varied by more than 10% from the mean weight) than when using the tablet splitter (3 out of 40 tablet halves). The dissolution and drug content of the hand-split tablets were therefore affected because of weight differences. However, the pharmacopoeia requirements for friability and disintegration time were met. Hand splitting of tablets can result in an inaccurate dose and may present clinical safety issues, especially for drugs with a narrow therapeutic window in which large fluctuations in drug concentrations are undesirable. It is recommended to use tablets with the exact desired dose, but if this is not an option, then a tablet splitter could be used.

## 1. Introduction

Tablet splitting is a known practice in inpatient and outpatient settings. Several studies studied the advantages of tablet splitting with regard to the health outcomes of patients, particularly within the geriatric and psychiatric communities. These advantages include dose adjustment [1], ease of swallowing and cost savings [2,3]. However, some studies revealed that splitting may result in the administration of an inaccurate dose, which can be of significant risk, especially if the split tablet formulation has a narrow therapeutic index [4,5]. Tablets are either hand-split or split using a variety of tablet splitters for scored or unscored tablets [6,7,8,9]. With these devices (which are easy to use), the tablets are cut into halves. Studies have also reported increased patient adherence due to ease of splitting, as well as cost benefits for the patient when a tablet splitter is used [6,8]. Some studies have found an impact of tablet splitting on the weight and content uniformity of the resulting tablet halves [10,11]. Elderly patients performing splitting may be of particular concern because of their reduced compliance and dexterity, i.e., ability to properly split the tablets [12]. Results from absorption and dissolution of split tablets have also shown that this population may be disproportionately affected [13,14]. When comparing hand splitting and tablet cutter procedures, a higher accuracy of tablet splitting was reported for tablet cutting devices versus hand splitting [15].

A review of clinical studies showed that tablet splitting may neither significantly affect clinical outcomes related to the management of hypertension, cholesterol or psychiatric disorders significantly nor influence overall patient adherence [3]. Lisinopril is an angiotensin converting enzyme (ACE) inhibitor drug with a half-life of 12 h. It is used to treat hypertension, congestive heart failure and heart attack, as well as, to prevent the renal and retinal complications of diabetes. Studies have suggested that splitting lisinopril tablets does not result in a significant change in blood pressure in patients with hypertension when comparing patients taking split lisinopril tablets versus whole lisinopril tablets [16]. However, this crossover study design on lisinopril did not indicate how the patients were instructed to split their tablets, nor did it address the individual outcome variations (i.e., only reported variations in the group outcomes). Moreover, each patient eventually self-administered one whole tablet because the two halves of the tablet were administered sequentially. Therefore, the study was unlikely to accurately reveal any potential clinical issues for splitting tablets. It has been documented that prescribing split tablets is not uncommon and represents a safety issue for the patient [17]. The recommendation has been to check the appropriateness of splitting tablets and use a pill-splitting device if necessary [18].

The purpose of our current study is to investigate the difference in the weight variation of the lisinopril split tablets (model drug) using two different tablet splitting methods and to analyse the impact of splitting lisinopril tablets on certain physical parameters such as the friability, dissolution and disintegration.

## 2. Materials and Methods

### 2.1. Materials

The lisinopril raw material sample was a gift from Neopharma Pharmaceutical Co., Abu Dhabi, UAE. Water, acetonitrile and methanol were obtained from Carlo Erba Reagent (Val de Reuil, France), and were of analytical grade. Potassium dihydrogen phosphate was purchased from Sigma Aldrich Co. (St. Louis, MI, USA). A tablet splitter was purchased from a local pharmacy.

### 2.2. Weight Variation of Lisinopril Tablets Before and After Splitting

Twenty whole tablets of lisinopril were weighed individually using a sensitive digital balance (±0.1 mg) (AUX Q1 220, Shimadzu, Japan). The average weight, standard deviation (SD), relative standard deviation (% RSD) and percentage difference of the weight of individual tablets compared to the average weight were calculated. The same weighed tablets were each cut by hand into two halves (right hand, 1st half and left hand, 2nd half) by a standard procedure, and each part was then weighed. Because one commercial product (i.e., tablets with the same size and shape) was chosen and one person was responsible for the hand splitting, confounding factors affecting the splitting of the tablets were minimized. The splitting procedure was performed by a right-handed person holding the tablet sides using the thumb on the top and the index finger at the bottom then pushing with two thumbs downward at the middle part of the tablet. This allowed for examination of any pattern regarding the weight of the resulting halves. A similar procedure was followed with the tablet splitter as previously detailed for the tablets allocated for hand splitting except that a splitter device was used instead of hand splitting. The United States Pharmacopoeia (USP) standards for weight variation test was applied for both whole and cut tablets [19]. Comparisons were made between the weight of the intact tablets and the corresponding total weight of the split tablets (paired *t*-test, α = 0.05) to determine if the splitting resulted in any significant loss of tablet mass.

### 2.3. Friability

Twenty tablets were randomly removed and brushed to remove any overlying dust and were weighed accurately. These tablets were subjected to a rotating drum of friability test apparatus (TA 220, Erweka, Heusenstamm, Germany). The drum was rotated at the speed of 25 rpm for 4 min. The tablets were de-dusted again and re-weighed. Percent friability was expressed by using the following formula:

$$\mathrm{Friability}\;=\frac{Initial\;Weight-Final\;Weight}{Initial\;Weight}\times 100$$

Ten tablets were split into halves using the same hand procedure as described in method 2.2. These halves were subjected to the same friability procedure described above.

### 2.4. Disintegration

A basket rack assembly was used (PTZ-Auto 02, Pharma Test, Hainburg, Germany) for the disintegration test in accordance with Appendix XII A of the 2013 British Pharmacopoeia (BP) [20]. One intact tablet was placed in each of the six tubes of the assembly. The assembly was suspended in the 1 litre beaker containing water and was observed during the complete disintegration period. The temperature was maintained at 37 °C throughout the experiment. The time of the initial and complete disintegration of the tablets was recorded.

Similarly, six new tablets were hand split into halves, and the above procedure was performed for both the 1st and 2nd halves. The time until complete disintegration for each group of halves was also noted.

### 2.5. Dissolution and In Vitro Drug Release

The release rate of lisinopril from the full tablets and the split tablets was determined using the DT 820 (Erweka, Heusenstamm, Germany). The dissolution test was performed in 900 mL of 0.1 N HCl at 37 ± 0.5 °C and 50 rpm in accordance with USP requirements for lisinopril tablets to ensure sinking conditions. A sample (10 mL) of the solution was withdrawn from the dissolution apparatus at 5, 10, 15, 30, 45, 60, 90 and 120 min. Each aliquot was replaced with fresh dissolution medium at the same temperature except for the final measurement. Each aliquot was filtered and diluted to a suitable concentration with 0.1 N HCl. The absorbance of these solutions was measured at 215 nm using the Venctron T70 double beam ultraviolet (UV) spectrophotometer (PG Instruments, Lutterworth, UK).

### 2.6. Spectrophotometric Analysis

The required sets of tablets/half-tablets were assayed by using double beam UV visible spectrophotometer (Venctron Type T70, PG Instruments, Lutterworth, UK). Twenty tablets of 20 mg lisinopril dihydrate (Zestril^®^ manufactured by AstraZeneca, Luton, UK) were crushed using a mortar and pestle. From the powdered tablets, a sample equivalent to 20 mg of lisinopril was weighed, dissolved in a 4:1 water to methanol solvent and diluted to obtain a concentration of 0.2 mg/mL. The resulting solution was then mixed well and sonicated for 5 min at room temperature. After sonication, the solution was filtered using 0.22 µm polypropylene membrane filters (Restek, Beijing, China) under vacuum (Rocker Scientific, New Taipei City, Taiwan). Serial dilutions with the solvent were prepared, and their absorbance was measured at a wavelength of 215 nm.

### 2.7. Statistical Analysis

SPSS, version 21 (SPSS Inc., Chicago, IL, USA) was used for descriptive and analytical statistics. For inferential statistical analysis, two-sided *t*-test analysis was used assuming *p* < 0.05. The figure preparation was conducted using Microsoft Excel (Microsoft Office 2010, Redmond, WA, USA).

## 3. Results and Discussion

### 3.1. Weight Variation of Lisinopril Tablets Before and After Hand Splitting

According to the USP standards for tablets [19], the requirements are met if (1) the weight of not more than two out of 20 tablets differs from the average weight by more 7.5% and (2) no tablet differs in weight by more than double that percentage. As such, the intact tablets were within the prescribed limits with an average weight of 0.22 g and 0.82% RSD. The maximum percentage difference from the mean for any tablet did not exceed 2.51% (Table 1). As a result of splitting, loss from the tablets to the surroundings was very small yet significant (*p* < 0.001) based on a paired *t*-test analysis. The maximum loss from any split tablet did not exceed 0.80%.

However, the 1st half of the split tablets had large variability with 15.83% RSD. Eleven out of the twenty (55%) halves had percentage differences from the mean exceeding 10%. Likewise, the 2nd half-tablet weight variability was high (10.14% RSD). Four out of 20 (20%) 2nd half-tablets exceeded the 10% difference from the mean. Moreover, the mean weight of the 1st and 2nd halves were significantly different from each other and from the corresponding expected ideal cut as indicated by the paired *t*-test analysis (*p* < 0.001).

When the individual 40 weights of the hand-split tablets were investigated for variability from the mean (mean = 0.11 g and 22.7% RSD), 37 out of 40 halves (92.5%) exceeded the 10% difference. These results are in agreement with a study involving eight frequently split medicines that have either narrow therapeutic indices or critical dosages, in which five of the studied drugs failed to meet the EP requirements for weight variations after hand splitting [21].

### 3.2. Weight Variation of Lisinopril Tablets Before and After Use of a Tablet Splitter

As with the tablets allocated for hand splitting, the intact tablets allocated for a tablet splitter fell within USP standards (Table 2). The maximum percentage difference from the mean for any of the tablets did not exceed 2.93%.

After splitting the tablets, the total weights of the pairs showed similar results for the mean and percentage RSD. None of the pairs’ weight deviated by more than 3.04% from the mean weight. The loss in tablet weights as a result splitting the tablets using the tablet splitter was small but significant (0.19% average weight loss) as indicated by the paired *t*-test analysis (*p* < 0.001). The tablet splitter showed lower weight loss compared to hand splitting technique; however, this should not be generalized because the results from another study on the narrow therapeutic window of levothyroxine showed higher fragmentation for tablets cut with a splitter compared to those cut with the hand [22].

**Figure 1:**
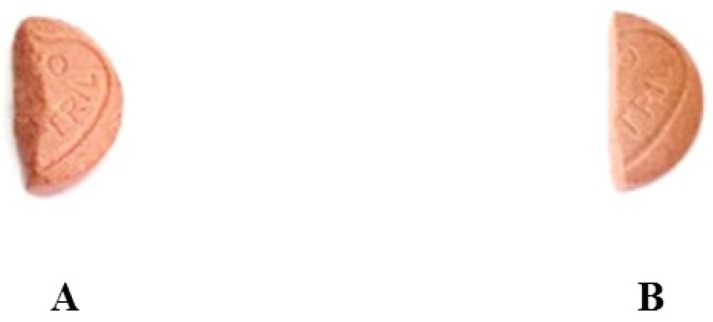
1st half of a tablet split by hand (**A**); and the smoother cut by a tablet splitter (**B**).

The variability in the weight of the 40 individual halves showed a mean of 0.11 g and 5.53% RSD with only three out of 40 halves (7.5%) differing from the mean by more than 10%. This is superior to the variability of the hand-split tablets and indicates that the use of tablet splitter resulted in more uniform weight because of the smoother cut as shown in Figure 1.

The results of the weight variations of lisinopril split tablets using the tablet cutter or hand splitting revealed that there was very small loss of mass. This finding is in agreement with other studies [15,23,24], and for drugs such as lisinopril that are taken over a long term, splitting is not expected to have significant clinical issues with medication that has long half-lives and large therapeutic windows [10].

However, a study of drugs with narrow therapeutic windows has shown that even with the presence of tablet scoring and Pharm. D. students performing the splitting with a knife, high variability in the weight could still be found [25]. The overall effect of tablet splitting on weight variation of lisinopril half-tablet is in agreement with a study on salbutamol tablets in which 15% of the half-tablets produced by the tablet splitter fell outside the USP acceptable range compared to 25% when hand splitting was used [15].

### 3.3. Friability

The results from friability testing showed that the friability of the intact tablets was 0.38% compared to 0.90% and 0.61% for the 1st and 2nd halves, respectively, which are altogether within the acceptable limits according to the BP. This indicates adequate tablet strength even after the initial stress due from breaking the tablets by hand. This strength may be the result of using an appropriate type and quantity of binding agent in the tablet formulation and using suitable compression force. The increased but acceptable rate of attrition from the half-tablets compared to the intact tablets can be explained by the uneven edges that result from splitting the tablets (see Figure 1.).

### 3.4. Disintegration

The average time noted for the initiation of disintegration process for whole, 1st half and 2nd half groups were 12, 9 and 6 s, respectively, whereas complete disintegration was achieved by 49, 39 and 36 s, respectively. The initial disintegration times correlate to the complete disintegration times and agree with the initial and final disintegration times observed in another study of capsules [26,27]. Because the BP requires that uncoated tablets should disintegrate within 15 min and film-coated capsules within 30 min, and USP specifies that uncoated and film-coated tablets should disintegrate within 30 min, both whole and hand-split tablets meet the pharmacopoeia requirements of the BP, USP and European Pharmacopoeia (EP) [19,20,28].

### 3.5. Dissolution and In Vitro Drug Release

**Figure 2:**
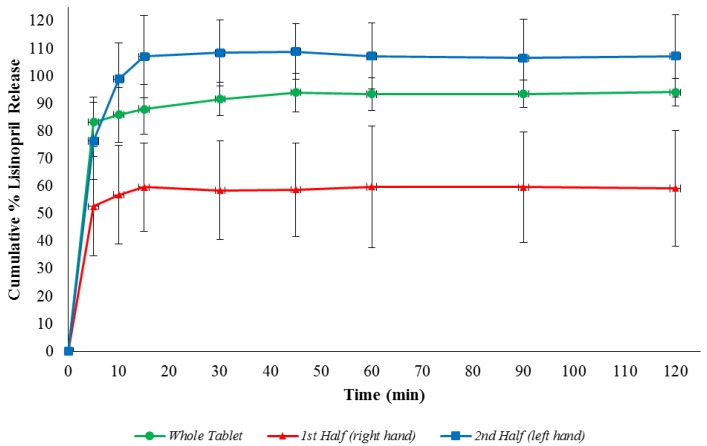
Two-hour dissolution profiles for intact, 1st half- (right hand) and 2nd half-tablets (left hand) of lisinopril calculated based on the labelled amount in 0.1 N HCl medium (*n* = 6). The error bars indicate the standard deviations.

The dissolution results for the whole lisinopril tablets and the dissolution of hand-split halves are shown in Figure 2. Most of the dissolution for the intact, 1st half- and 2nd half-tablets occurred within the first 5 min, which is in accordance with the disintegration results. The percentage released from the intact tablet was greater than 91.6% after 30 min. This corresponded to 58.5% of the 1st half and greater than 108.4% of the 2nd half hand-split tablets. Although the dissolution profile of the 2nd half-tablets did not differ significantly from the intact tablets, the 1st half-tablets showed significant differences over time extending from 10 min onward compared to both 2nd half-tablets and intact tablets as shown in Figure 2. This can simply be attributed to the consistently lower weights of the 1st half (right hand) compared to the 2nd half rather than intrinsic dissolution behaviour. This is concomitant with another study on levothyroxine in which the dissolution of split half-tablets conformed with content uniformity [22].

The dissolution requirements according the USP and BP is that the amount of drug released (dissolution) should not be less than 80% of the labelled amount at 30 min. The intact tablets and the 2nd half-tablets met the dissolution requirement but not the 1st half-tablets.

### 3.6. Lisinopril Spectrophotometric Assay

According to the USP and BP, lisinopril tablets should not contain less than 90% and not more than 110% of the labelled amount. The analysis of the content of the whole tablets using UV was 103.9% of the labelled amount. This corresponds to a mass of 20.78 mg. The results from the hand-split tablets did not fully comply with the requirements. Assuming that the labelled amount is half of the original labelled amount (i.e., 10 mg), the 1st half-tablet had 92.8% of the expected drug content whereas the 2nd half-tablet had 124.0%. Although the result from the 2nd half-tablet did not pass the pharmacopoeia requirements, the 1st half-tablet passed just at the lower margin of the acceptable range. The results are in accordance with another study investigating the content uniformity for 16 commonly used medications prescribed in an outpatient setting when split by a knife [10]. Our current results indicate that although taking each split half sequentially will not affect the average steady state concentration, the impact on the fluctuation between maximum and minimum concentrations is considerable. The results of the UV visible spectrophotometric analysis for lisinopril showed that the drug concentrations were linear in the range of 4–32 μg/mL, and the correlation coefficient value of 0.9993 indicates that developed method was linear. The limit of detection and limit of quantitation values were 0.8155 μg/mL and 2.5790 μg/mL, respectively.

Splitting lisinopril tablets (Zestril^®^ tablets, 20 mg) either by hand or using a tablet splitter did not influence the friability and disintegration. However, splitting tablets can significantly affect the weight of the resulting halves, particularly when using hands to perform the splitting. The potency of the hand-split tablets was also affected in concordance with the weight variation. Therefore, although the dissolution behaviour was similar for the intact and split tablets, the percentage of the drug dissolved at different times was higher with the 2nd half of the split tablets compared to the 1st half, which reflects its greater weight and hence increased chemical content. The impact of this may or may not be of clinical importance. Although hand splitting of tablets for drugs that have narrow therapeutic windows can result in an unacceptable fluctuation in blood concentrations, the long-term use of split-tablet drugs with long half-lives and wide therapeutic windows may not be associated with clinical difference compared to the use of intact tablets comprising the required dose. Fragment loss to the surroundings using both splitting methods was quite small and should not pose any clinical issue for Zestril^®^ tablets. Nevertheless, it is recommended to use either the exact tablet dose when possible or a tablet splitter device when only double the required dose is commercially available.

## Figures and Tables

**Table 1:** Weight results for lisinopril tablets before and after hand splitting (*n* = 20).

Analysis	Intact Tablets	Sum of the Two Halves	% Weight Loss	1st Half (Right Hand)	2nd Half (Left Hand)
Mean (g)	0.2249	0.2245	0.2178	0.0911	0.1334
SD	0.0018	0.0018	0.1833	0.0144	0.0135
RSD (%)	0.8219	0.8240	-	15.8318	10.1434
Maximum Difference (%)	2.5139	2.4704	0.7979	37.2119	−25.3871

**Table 2:** Weight results for lisinopril tablets before and after splitting using the tablet splitter (*n* = 20).

	Intact Tablets	Sum of the Two Halves	% Weight Loss	1st Half	2nd Half
Mean (g)	0.2235	0.2230	0.1924	0.1148	0.1083
SD	0.0020	0.0020	0.0858	0.0052	0.0051
RSD (%)	0.8884	0.8853	-	4.5188	4.7066
Maximum Difference (%)	2.9313	3.0401	0.3586	−9.2058	10.2951
